# Modified Enzyme Substrates for the Detection of Bacteria: A Review

**DOI:** 10.3390/molecules25163690

**Published:** 2020-08-13

**Authors:** Laura Pala, Teja Sirec, Urs Spitz

**Affiliations:** Biosynth Carbosynth, Axis House, High Street, Compton, Berkshire RG20 6NL, UK; laura.pala@biosynth-carbosynth.com (L.P.); teja.sirec@biosynth-carbosynth.com (T.S.)

**Keywords:** enzyme substrates, bacterial detection, chromogenic substrates, fluorogenic substrates, luminogenic substrates, redox substrates

## Abstract

The ability to detect, identify and quantify bacteria is crucial in clinical diagnostics, environmental testing, food security settings and in microbiology research. Recently, the threat of multidrug-resistant bacterial pathogens pushed the global scientific community to develop fast, reliable, specific and affordable methods to detect bacterial species. The use of synthetically modified enzyme substrates is a convenient approach to detect bacteria in a specific, economic and rapid manner. The method is based on the use of specific enzyme substrates for a given bacterial marker enzyme, conjugated to a signalogenic moiety. Following enzymatic reaction, the signalophor is released from the synthetic substrate, generating a specific and measurable signal. Several types of signalophors have been described and are defined by the type of signal they generate, such as chromogenic, fluorogenic, luminogenic, electrogenic and redox. Signalophors are further subdivided into groups based on their solubility in water, which is key in defining their application on solid or liquid media for bacterial culturing. This comprehensive review describes synthetic enzyme substrates and their applications for bacterial detection, showing their mechanism of action and their synthetic routes.

## 1. Introduction

The ability to detect and identify bacterial species is of paramount importance in clinical diagnostics, environmental testing, food security settings, as well as in microbiology research [1,2,3,4]. The development of methods for bacterial detection has evolved over the last 70 years. Healthcare threats such as the occurrence of multidrug-resistant bacterial pathogens motivated the global scientific community to develop methods for bacterial detection that are rapid, specific and economically viable [1,2,3,4].

**Figure 1:**
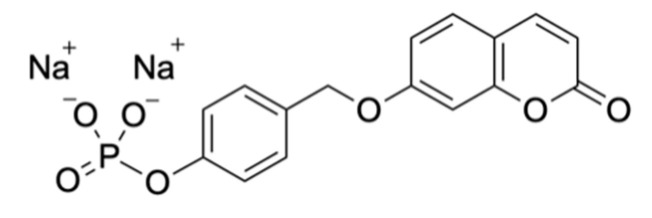
Typical synthetic enzyme substrate structure.

The methods for bacterial identification predominantly detect the bacterial “fingerprint” based on their genotype (nucleic acid sequences) or phenotype (antigens or enzymes). While both approaches find applications in diagnostic clinical microbiology, the PCR-based molecular method based on the detection of bacterial genotype has been extensively used in recent times. Even though this molecular method is powerful, it presents with some limitations that should be addressed by novel methods. For example, as PCR is based on the detection of species-specific sequences of nucleic acids, this method does not give any indication on whether the given pathogen is viable or dead. Moreover, PCR identifies the presence of a gene but gives no indication whether it is expressed in a certain bacterial sample. Thirdly, in order to perform a PCR, the nucleotide sequence of a desired marker gene should be known in order to properly design the oligonucleotides (primers) needed for this method. The latter point might be a limitation in new clinical isolates, as bacteria can acquire or lose genetic elements or undergo genetic rearrangements [5,6]. On the other hand, enzyme-based detection methods also have their own limitations, one of them being need for culturing, which can take from several hours to days. Comparison of the molecular and phenotypic methods for bacterial detection was extensively discussed in a recent review [7]. This review focuses on the method for identification of bacteria based on the use of synthetic enzyme substrates, which allow for the detection of a species’ specific enzymatic activity [1,2,3,4]. Herein, a synthetic substrate, specific for a marker enzyme in a specific bacterial species, is added to a bacterial culture in liquid or solid growth media. A synthetic enzyme substrate consists of the following chemical substructures: (a) the enzyme labile group, which is recognized by the target enzyme and is subsequently utilised during enzymatic reaction, (b) the signalogenic substructure, which produces a detectable signal after the enzymatic reaction and (c) the labile spacer, which is an optional substructure utilized to eliminate undesirable interactions such as susceptibility to non-enzymatic cleavage of the labile bond by spontaneous hydrolysis in the medium resulting in undesirable background signals. Figure 1 shows an example of a typical synthetic enzyme substrate substructure. The phosphate group acts as an enzyme labile group and serves as a substrate for phosphatase enzymes. The *p*-hydroxybenzyl alcohol acts as a self-immolative spacer, which, after enzymatic reaction, generates a quinone methide by spontaneous 1,6-elimination [8,9]. The fluorescent reporter 4-methylumbelliferone moiety acts as the signalogen.

The main advantages of synthetic enzyme substrates for the detection of bacteria are their ease of use, stability in culture media, relatively low price and flexibility in their design [1,2,3,4].

Over the last 70 years, this field advanced with the development of synthetic substrates generating detectable signals upon specific enzymatic reactions. This allows for the identification of microorganisms, even in primary isolation medium, thus circumventing the need for subculture and further biochemical tests to establish their identity [10]. Marker enzymatic reactions in microorganisms are used as unique targets for identification and/or differentiation purposes, involving respective substrates or their analogues [1,2,3,4,11]. In addition to the detection of specific microbial species, enzyme substrate-based approaches to detection can provide insights into the bacterial metabolic state, for example, in the event of an invasion of pathogens into host cells. Enzymes that are differentially expressed at various stages of pathogenesis can serve as the biomarker’s indication of a certain state of metabolism, such as the state of virulence of a microbial organism. A state of virulence may be triggered by environmental factors, such as temperature or certain chemicals, inducing gene expression from mobile genetic elements, for example, bacterial virulence plasmids, which provide the organism with the capability of host invasion [12]. Therefore, enzyme substrates can serve to detect virulence factors and other enzymatic markers of a bacteria’s metabolic state.

Synthetic enzyme substrates are used in clinical microbiology, environmental testing, food security and microbiology research [1,2,3,4]. This review presents examples of applications for each subtype of enzyme substrate and reviews enzyme substrates according to two criteria used for classification: the type of signal generated (fluorogenic, chromogenic, luminogenic and electrogenic) and the type of target enzyme that triggers the signal (hydrolytic and non-hydrolytic). 

## 2. Classification of Synthetic Enzyme Substrates

In this review, enzyme substrates are discussed according to their classification, thus providing a rationale to design the most suitable enzyme substrate for a specific application. In addition, this review presents an overview of signalogenic structures of synthetic enzyme substrates for a range of specific signal detection methods. This review further describes synthetic routes and challenges in synthesis of modern enzyme substrates. 

Signalogenic substructures producing a detectable signal are classified into chromogenic, fluorogenic, luminogenic and electrogenic (Table 1).

### 2.1. Classification by Type of Generated Signal

#### 2.1.1. Chromogenic and Fluorogenic Substrates

Chromogenic and fluorogenic substrates will be discussed in the same chapter, as their principle of action is common. For both types, an enzyme substrate is linked to a signalophor, which, upon enzymatic cleavage, is released from the molecule and a detectable signal is generated. At this point, classification of the chromogenic and fluorogenic substrates by the type of hydrolysis product they form, whether it is soluble and non-precipitating or insoluble and precipitating, is implemented. This is an important distinction, as it defines the applications. It is essential to highlight that, in this review, the terms soluble/non-precipitating and insoluble/precipitating enzyme substrates are not referring to the solubility of the enzyme substrate itself, but rather to the solubility of the released signalophor following enzymatic cleavage. Moreover, we are referring to solubility in water as this solvent is the key component in biological systems.

The non-precipitating/soluble enzyme substrates release a signalophor suitable for use in liquid media. Such assays are well suited to measure signalophor concentrations, binding constants and enzyme kinetics. On the other hand, this type of substrates is not ideal for use in solid microbiological media, as a soluble signalophor can rapidly diffuse across the agar plate, thus the positive colonies are difficult to identify. 

Insoluble/precipitating substrates are well suited for use in solid media, as they produce a signalophor that is insoluble in water. This is particularly advantageous as it cannot diffuse through the medium and therefore provides information regarding the physical location of the enzymatic reaction. This is important for the detection, isolation and differentiation of individual colonies from a mixed culture in one single assay.

##### Soluble Substrates

**Figure 2:**
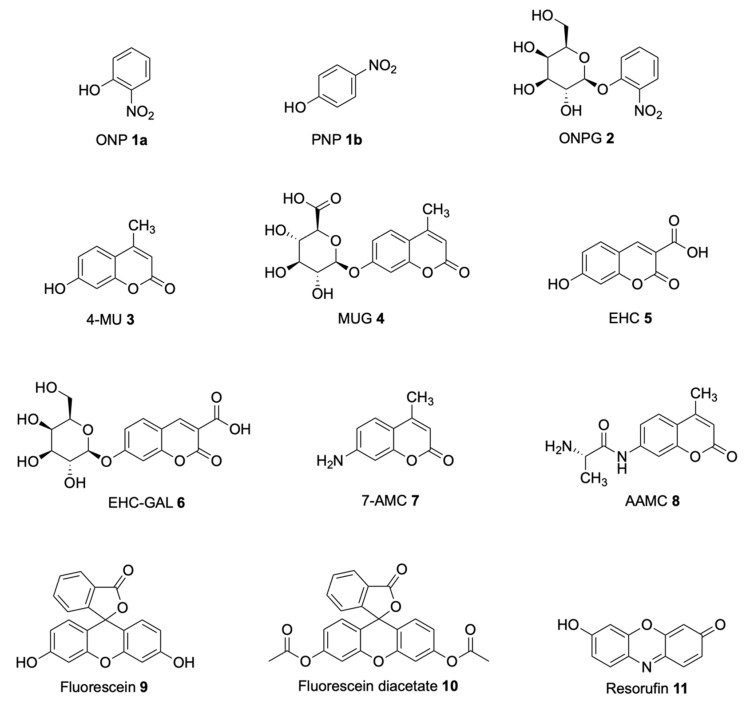
Chemical structures of signalophors (**1**,**3**,**5**,**7**–**9**,**11**) and of modified enzyme substrates (**2**,**4**,**6**,**10**).

The most established soluble synthetic enzyme substrates are conjugates of *o*-nitrophenol (ONP) **1a** or *p*-nitrophenol (PNP) **1b** (Figure 2), discovered in 1939 and 1950 by Aizawa and Lederberg. The first synthetic substrate was a chromogenic β-d-galactosyl derivative of nitrophenol (*o*-nitrophenyl β-D-galactoside, ONPG) **2** (Figure 2) and was used for the detection of *E. coli* via its β-galactosidase activity [13,14]. More conjugates of ONP and PNP were synthesized to detect the presence of other marker enzymes in bacteria. The signalophors ONP and PNP that get released by the enzymatic reaction are soluble, develop a yellow colour, and are detectable spectrophotometrically at A_405_, particularly when present in their phenolate form in aqueous solutions [15]. While the use of these chromogenic substrates was fundamental to microbiology research in the second half of the 20th century, their use in diagnostic applications was limited, since the yellow colour of the signalophor is overlapping with the colour of bacterial culture media [16].

Several coumarin derivatives conjugated with an enzyme substrate are used to detect bacteria, thanks to the fluorescent signalophor released by the enzymatic cleavage. One of the most widely used coumarin derivatives is 4-methylumbelliferone (4-MU) **3** (Figure 2).

Derivatives of 4-MU were first used by Dyer to detect bacterial enzymes [17]. 4-MU is conjugated to specific enzyme substrates to detect bacterial glycosidases and phosphatases in liquid assays, producing a strong fluorescence signal after cleavage of the synthetic 4-MU-conjugate. The fluorescent signal can be quantified fluorometrically at an emission wavelength of 442 nm, upon excitation at 366 nm [15,17]. 4-Methylumbelliferyl-β-d-glucuronide (MUG) **4** (Figure 2) is an example of a 4-MU conjugate widely used for the detection and quantification of β-D-glucuronidase (GUD) positive *E. coli* in drinking and wastewater monitoring [18,19].

However, the use of 4-MU-conjugates in living bacteria presents some limitations; for example, 4-MU esters show a limited stability in water, and consequently assays must be carried out within a short time. Additionally, 4-MU appears blue under UV light, which overlaps with the natural fluorescence emission of many microbiological media, leading to high background fluorescence that interferes with signal detection. Finally, the 4-MU fluorescent signal is best detected at biologically incompatible pH values of 10–11 because only the deprotonated form of 4-MU exhibits strong fluorescence [11]. 

Other coumarin derivatives have been investigated in order to overcome the drawbacks associated with 4-MU. Substrates based on the ethyl 7-hydroxycoumarin-3-carboxylate (EHC) **5** (Figure 2) core show increased fluorescence at a broader pH range and reduced toxicity. The enzyme substrate EHC-GAL **6** (Figure 2) was used in the detection of coliforms achieving bacterial detection that was 1 h faster compared to 4-MU-GAL [20].

7-Amino-4-methyl coumarin (7-AMC) **7** (Figure 2), like 4-MU, is a water-soluble coumarin derivative producing strong blue fluorescence which is measured at similar excitation and emission wavelength (excitation wavelength of 365 nm and an emission wavelength of 440 nm) [21]. In contrast to 4-MU, 7-AMC contains an amino group allowing for the generation of peptidase substrates [10,22]. Another important advantage of AMC conjugates over their MU relatives is that they can be conveniently used at a physiological pH. Although their emission does decrease at low pH, their useful pH range is nevertheless broader. L-alanine-7-amido-4-methylcoumarin (AAMC) **8** (Figure 2) is used as the substrate for L-alanylaminopeptidase for the reliable detection of Gram-negative bacteria, as this aminopeptidase is absent in Gram-positive bacteria. In addition, AAMC inhibits the growth of these non-target organisms on agar plates [23].

Fluorescein-based enzyme substrates are another common class of fluorescent probes in life sciences thanks to their sensitivity, brightness, biocompatibility, excitation (485 nm) and emission (514 nm), which are in the visible range of the light spectrum [24,25]. For example, Fluorescein **9** (Figure 2) is a dark orange/red coloured synthetic fluorescent signalophor that emits green light when excited with blue light. Fluorescein suffers from intrinsic hydrophilicity, which limits its use for bacterial detection on agar plates [24], and low photostability, leading to significant photobleaching via oxidative degradation of the fluorophore in its excited state [26]. Similar to other types of fluorogenic substrates, the ultimate structure of the fluorescein-based synthetic substrate dictates its permeability, cellular localization, intracellular aggregation and interconversion between the fluorescent “open form” and the non-fluorescent spirocyclic “closed form” [25,26,27].

Fluorescein diacetate **10** (Figure 2), a nonfluorescent derivative of fluorescein, has been used in a viability assay for the detection of metabolically active *M. tuberculosis* in sputum [28] and for the detection of bacteria in wastewater [29]. Fluorescein diacetate enters living bacterial cells and undergoes deacetylation by nonspecific esterases, resulting in the release of fluorescein, which accumulates within cells and allows for the direct visualization of living cells by epifluorescent microscopy. Fluorescein-conjugated vancomycin has also been used in a non-invasive approach for diagnosis of infections with Gram-positive bacteria [30]. Breeuwer et al. applied fluorescein-conjugates, 5(6)-carboxyfluorescein succinimidyl esters, for monitoring cytoplasmic pH changes in *E. coli* [31].

Resorufin **11** (Figure 2) is a water-soluble signalophore with chromogenic and fluorogenic properties, when used in synthetic enzyme substrates. It can be measured by excitation at 574 nm and emission at 581 nm, which is within the visible range and thus broadens its applicability by enabling detection on most plate readers, gel imagers and standard fluorescence microscopes [32]. Resorufin has a high extinction efficient (ε = 51,000 at 572 nm), producing a bright pink colour visible to the naked eye, even at very low concentrations. The pink colour is associated with the deprotonated form, which predominates over the protonated form (orange) under physiological conditions, thanks to its acidic properties. In fact, its pK_a_ of 5.8 is lower than the pK_a_s of both *p*-nitrophenol and 4-MU (7.2 and 7.8, respectively), resulting in increased sensitivity. This is particularly advantageous when resorufin-based substrates are used for bacterial detection, where lower amounts of substrate are required [33]. Alkylated and glycosylated conjugates of resorufin are used extensively as enzyme substrates for esterases and glycosidases [34,35,36].

**Scheme 1:**
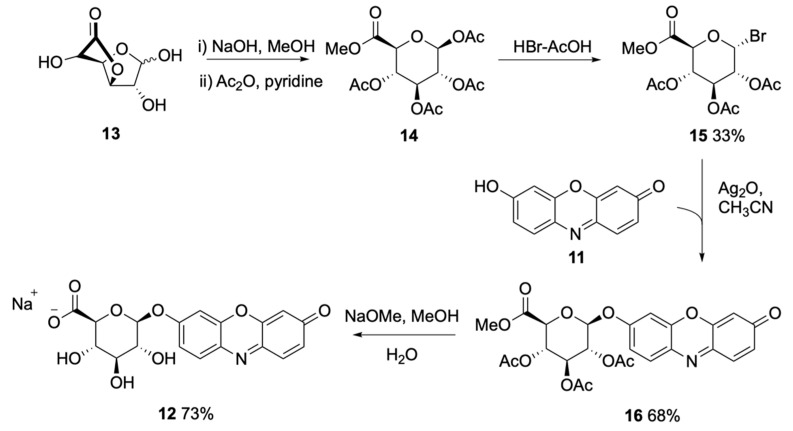
Recently proposed synthetic route to obtain enzyme substrate resorufin-β-glucuronide (REG) **12** [33].

Resorufin-β-glucuronide (REG) **12** (Scheme 1) has been used as a substrate for β-D-glucuronidase, an enzyme occurring almost exclusively in *E. coli*, for the detection of *E. coli* in drinking water. This simple, fast and sensitive test offers a promising alternative to MUG [33]. A notable advantage of REG over MUG is that a UV source is not required for the analysis, making the REG chromogenic substrate appealing when equipment availability is limited. Unfortunately, the high cost of REG ultimately prevented its widespread use in water quality testing. To overcome this, the authors proposed a convenient easily scalable 4-steps synthetic route for REG (Scheme 1), using inexpensive and available starting materials and avoiding expensive procedures such as chromatography. By treating d-glucurono-6,3-lactone **13** with sodium methoxide and successive acetylation, glucopyranuronate **14** is obtained. Bromination with HBr results in glucopyranuronate **15**, which reacts with resorufin **11** using a modified Koenigs−Knorr method to form protected resorufin glucuronide **16**. The final step of methanolysis and hydrolysis generates REG **12** [33].

Recently, resorufin-β-d-glucuronide methyl ester (RUG^TM^), the methyl ester analogue of REG, was reported as a cheaper alternative (250 times less expensive), with comparable performance to REG in the detection of *E. coli* in quality water testing [37]. The unspecific hydrolysis of the methyl ester in RUG generates REG, which undergoes the previously described enzymatic reaction to form bright pink resorufin. RUG has successfully been used in a simple chromogenic test kit for developing countries [37].

**Scheme 2:**
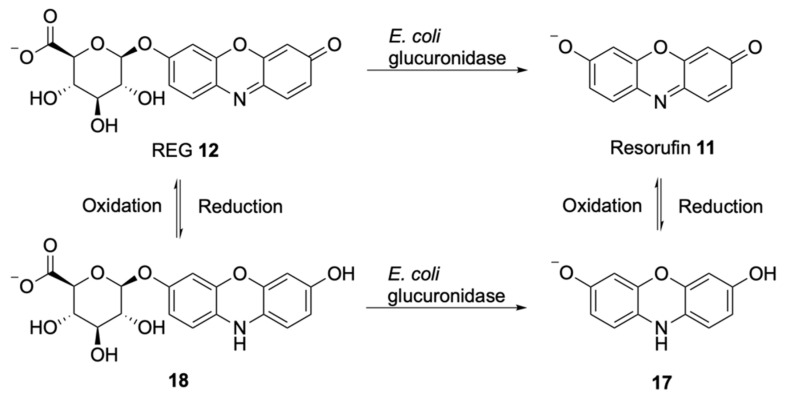
Undesired reduction of REG **12** and resorufin **11** in absence of oxygen, generating colourless dihydroresorufin **17** and REG dihydroresorufin derivative **18** [33,37].

The only drawback of REG and RUG is the required presence of oxygen in order to prevent the reduction of resorufin to dihydroresorufin **17** and of REG to its dihydroresorufin derivative **18**, as reported in Scheme 2, which causes bleaching and consequent decreased intensity in colour necessary for detection. This limits the usage to well-aerated cultivation systems, or it can be used in the detection of oxygen, done under anaerobic cultivation conditions [33,37].

##### Insoluble Substrates

Due to the nature of microbiological analysis in diagnostic testing, there was a need to develop synthetic enzyme substrates suitable for the use in solid microbiological media. Several approaches were used to overcome the issue of the diffusion of colour in agar plates.

Aromatic derivatives containing two hydroxyl groups in *ortho* position to each other conjugated to enzyme substrates were widely described in the literature [38,39,40,41,42]. Following enzymatic cleavage, the phenoxide forms, precipitating coloured chelates in the presence of metal ions added to the culture media. Examples of such probes are glycosylated versions of cyclohexenoesculetin and alizarin [38,39,40,41,42].

**Figure 3:**
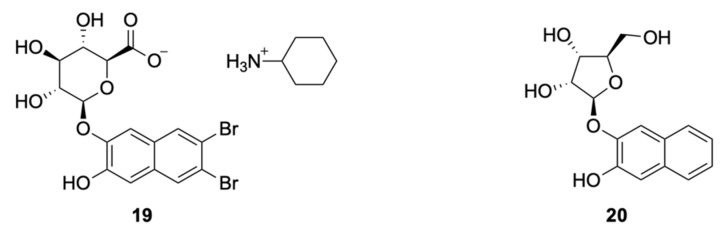
Structures of the β-d-glucuronide derivative **19** and the β-d-riboside derivative **20** [43].

Recently, a series of insoluble chromogenic enzyme substrates for bacterial glycosidase detection were introduced, based on a derivative of the metal chelator catechol 2,3-dihydroxynaphtalene. The newly reported 2,3-dihydroxynaphtalene derivatives are easily prepared from inexpensive and readily available 2,3-dihydroxynaphtalene and are bromo-substituted in the 6 and 7 positions. The presence of the bromo substituents causes a decrease in solubility of the resulting chelated metal complexes, and consequently minimizes the diffusion into the agar media. For this reason, these newly developed substrates are effective insoluble chromogenic substrates in agar media [43]. An example is the β-d-glucuronide derivative **19** (Figure 3), which is effective in the detection of *E. coli* on agar plates, producing an intense brown colour in the presence of ammonium iron (III) citrate. Interestingly, the non-halogenated versions of the above-mentioned 2,3-dihydroxynaphtalene derivatives are water soluble and are effective as soluble chromogenic enzyme substrates for bacteria detection. An example is the β-d-riboside derivative **20** (Figure 3), effective in the detection of *S. aureus* [43].

The most successful strategy to generate colony-restricted coloration was the use of indoxyl substrates. In addition to the advantage of water-insoluble dyes, differently substituted indoxyl substrates produce a range of different colours. Having a choice of colours when using chromogenic indicator systems is highly desired, allowing for the detection of two or more enzymes in parallel or in applications that require optical contrast against an off-white background [11]. Thanks to the precipitating nature of the signalophor, indoxyl-based substrates have greatly broadened the application of chromogenic substrates in microbiology [44].

**Scheme 3:**
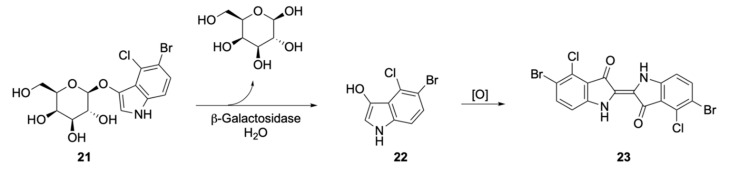
Indigo dye formation using indoxyl substrate X-Gal.

**Figure 4:**
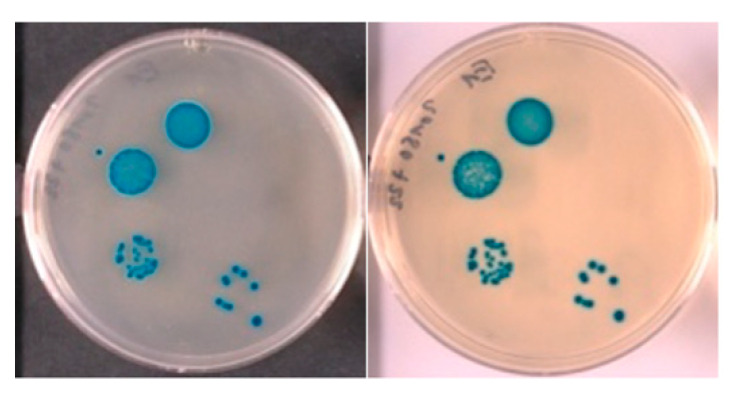
Colonies of *E. coli* on a plate containing X-Gal. Pictures kindly supplied by Dr Julian Ihssen, Biosynth Carbosynth.

The revolutionary invention of indoxyl substrates in the 1950s (primarily for histochemistry) also quickly gained popularity for bacterial detection [11]. The enzyme labile group is attached to an indoxyl moiety, which is then cleaved by a specific enzyme. The released indoxyl signalophor undergoes oxidation and dimerization via the typical indigo reaction in the presence of oxygen to result in an intensely coloured product [11,44]. In addition, unlike the initial indoxyl substrate, the formed indigo dye is insoluble in aqueous media, thus facilitating the visualization of bacterial colonies on agar plates and revealing where the enzymatic cleavage takes place [44]. An example is shown in Scheme 3, where 5-bromo-4-chloro-3-indoxyl- β-d-galactopyranoside **21**, also known as X-Gal, is used as a chromogenic indicator for the detection of β galactosidase activity. The enzymatic cleavage of the substrate generates 5-bromo-4-chloro-3-hydroxyindole **22** and the blue indigo product **23**, which can be easily detected on agar plates. X-Gal is used for the detection of *E. coli*, as shown in Figure 4, and other β-galactosidase-positive enterobacteria (so-called coliforms), differentiating these species from β-galactosidase-negative bacteria such as *Salmonella enterica* and *Pseudomonas aeruginosa*.

Typical enzyme labile groups attached to the indoxyl moiety are fatty acids linked via an ester bond. These include C-8 esters, which are useful in detecting the esterase activity of various *Salmonella* strains. Other examples are carbohydrates linked via a glycosidic bond to the indoxyl moiety, for example, glucosyl or galactosyl derivatives, that allow for the detection of specific groups of bacteria, such as *Enterobacter* spp. or coliforms [44].

Further improvements and customization were achieved by synthesizing indoxyl variants with different halogenation patterns. These have a dramatic effect on the colour and intensity of the respective chromogens [10]. For example, 5-bromo-4-chloro-indoxyl will form a bright green-blue dye, 5-bromo-6-chloro-indoxyl a magenta dye, *N*-methylindolxyl a green dye, 6-chloro-3-indoxyl a salmon orange dye and bromo-3-indoxyl substrates a lapis coloured dye. These derivatives offer excellent colour flexibility and are widely used in primary selective media to conduct bacterial enumeration and detection directly on the isolation plate [10]. Indoxyl glycosides, including glucoside, galactoside and glucuronide derivatives, are widely used because of their high sensitivity, low toxicity and availability from a number of commercial sources [45]. However, in spite of the wide applications of indoxyl-based substrates for microbial detection/differentiation, toxicity against some microbial species was also reported [46].

**Figure 5:**
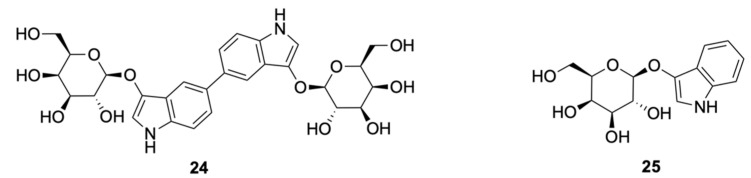
Structures of polymeric indoxyl substrate **24** and 3-indoxyl-β-d-galactopyranoside **25**.

**Figure 6:**
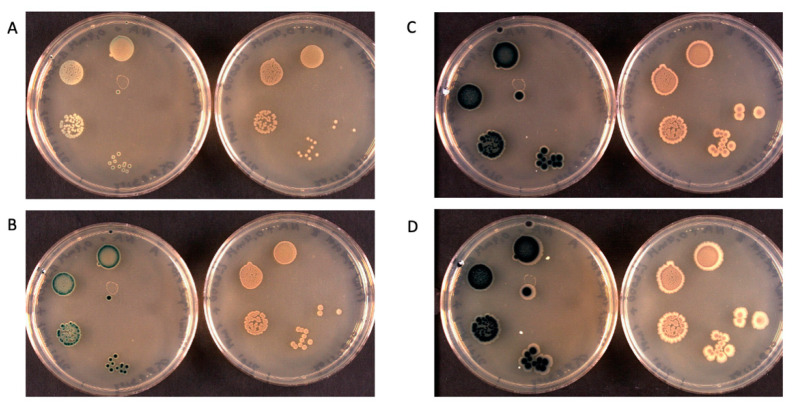
Test on agar plates (nutrient agar, 5 g/L peptone, 5 g/L NaCl, 2 g/L yeast extract, 1 g/L meat extract, 15 g/L agar, pH 7.4). Inducer for β-galactosidase: IPTG, 1 mM. Test strain: *E. coli* ATCC 25922 (β-galactosidase positive). Bacteria were incubated in presence of indoxyl substrate **24** (left) or **25** (right) for 16 h (**A**), 24 h (**B**), 48 h (**C**), 6 days (**D**). Pictures kindly supplied by Dr Julian Ihssen, Biosynth Carbosynth.

In order to improve the properties of the indigo derivatives generated by the enzymatic reaction, polymeric versions of indoxyl enzyme substrates were investigated by Biosynth Carbosynth (data not published). In this design, the two indoxyl moieties of dimeric substrates are connected via C-C linking between the phenyl groups, and each of the indoxyl groups is bonded to an enzyme substrate. The presence of the bacteria of interest causes enzymatic cleavage of the two substrates. Then, oxidative coupling takes place, generating the typical indigo unit at both ends, in a polymeric chain. An interesting property of polymeric indigo derivatives is their improved chromogenic properties, which were investigated and described in previous work [47,48]. An example of such a polymeric substrate is B-3000 or 3,3′-bis(β-d-galactopyranosyl)-5,5′-bi-1H-indol **24** (Figure 5), which was tested against I-6000, 3-indoxyl-β-D-galactopyranoside **25** (Figure 5) in the detection of *E. coli* on agar plate, showing a blue colour after 24 h, which became almost black after 48 h (Figure 6) (data not published). These results show that polymeric indoxyl enzyme substrates are a promising alternative to the commonly used indoxyl substrates, due to their improved chromogenic properties.

The strategy used for the indoxyl substrates was further developed in the design of a series of probes commercialised by Biosynth Carbosynth as Aldol^®^ substrates [49]. These present several advantages: they are useable in the absence of oxygen or oxidisers, have a tuneable colour range broader than commonly used chromogenic substrates, have both chromogenic and fluorogenic properties, and have insoluble signalophors (with the exception of Aldol^®^ 458, which is water soluble at pH ≥ 6).

**Scheme 4:**
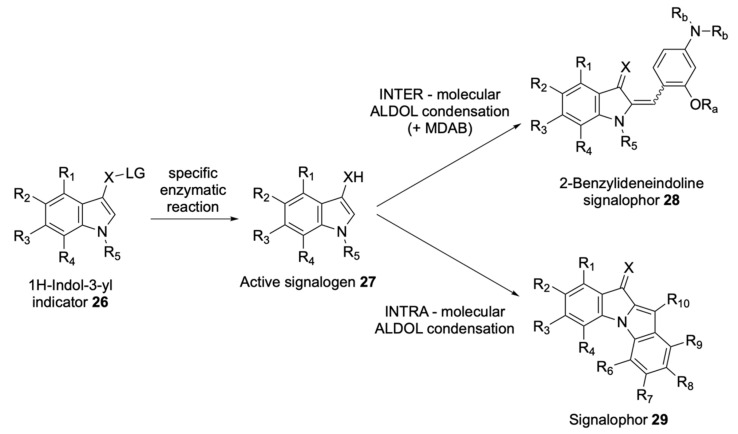
Mechanism of action of general Aldol^®^ substrates to form signalophor **28** when an inter- molecular aldol condensation occurs, and signalophor **29** in case of intra-molecular aldol condensation [49].

The mechanism of action of Aldol^®^-conjugated enzyme substrates (Scheme 4) is based on an inter- or intra-molecular aldol condensation between an aldol donor group, whose precursor is a generic 1H-indol-3-yl indicator **26**, and an acceptor. Following specific enzymatic cleavage, the aldol donor group is released and the active signalogen **27** is generated. **27** reacts with the acceptor in the aldol condensation, generating a coloured and/or fluorescent species, and the detection and/or quantification of which relates to the presence of the investigated pathogen [49]. The acceptor can be added as an auxiliary reagent, such as 2-methoxy-4-(*N*,*N*-dimethylamino)benzaldehyde (MDAB), leading to an inter-molecular aldol condensation forming a signalophor species with the general structure of 2-benzylideneindoline **28**. Alternatively, the acceptor can be conjugated to the donor, allowing an intra-molecular aldol condensation to form a 10H-indolo [1,2-β] indole derivative **29** [49]. Biosynth Carbosynth developed a series of Aldol^®^ substrates with different properties as summarized in Table 2.

**Scheme 5:**
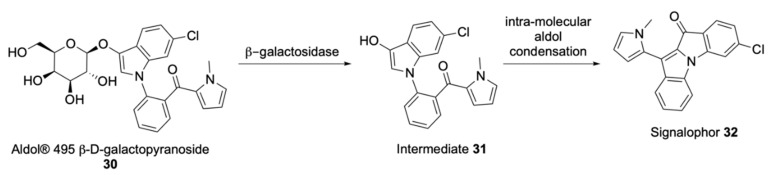
Enzymatic cleavage of Aldol^®^ 495 β-d-galactopyranoside **30** generates intermediate **31**, which undergoes intra-molecular aldol condensation forming insoluble orange signalophor **32**.

An example of a substrate generating a signalophor via an intra-molecular aldol condensation is Aldol^®^ 495 β-d-galactopyranoside **30** (Scheme 5), used as a chromo- and fluorogenic indicator for β-galactosidase activity. The β-d-galactopyranose moiety is cleaved by bacterial β-galactosidase, generating the intermediate **31** acting as aldol donor and containing the aldol acceptor incorporated into the same molecule. Upon intra-molecular aldol condensation, the insoluble orange product **32** is generated in liquid media or agar plates (Scheme 5). In the presence of a fluorescence enhancer or a suitable matrix, green fluorescence signals are observed. Importantly, the aldol condensation proceeds in the presence and absence of oxygen, making the probe suitable for both aerobic and anaerobic bacterial cultures [49].

**Figure 7:**
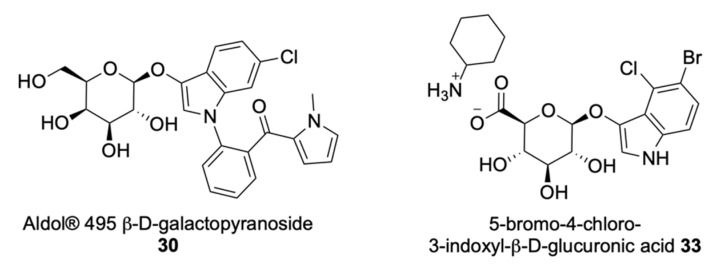
Structures of substrates **30** and **33**.

**Figure 8:**
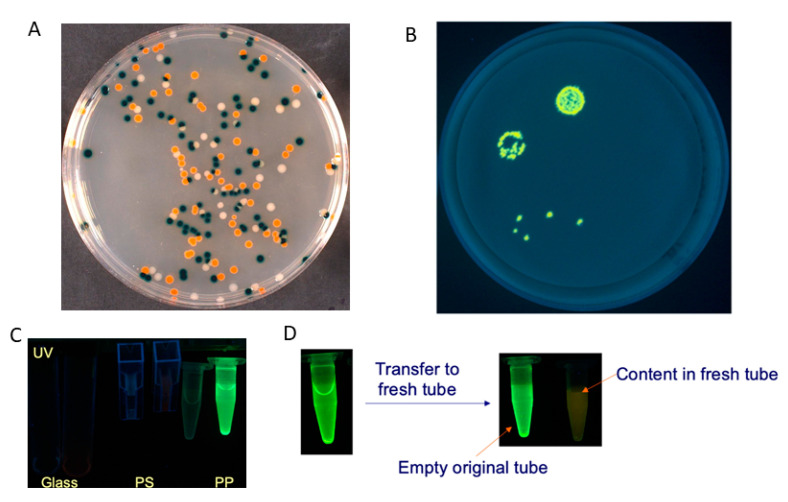
Mixture of *Klebsiella pneumoniae* (β-galactosidase positive and β-glucuronidase negative, orange colour), *E. coli* (β-galactosidase positive and β-glucuronidase positive, dark-violet colour), and *Salmonella enteritidis* (negative for both enzymatic activities, uncoloured) on a single plate after 24 h of incubation (**A**). Detection on membrane filter of *Clostridium perfringens* cultivated under anaerobic conditions by using Aldol^®^ 495 β-D-galactopyranoside **30** (**B**). Fluorescent emission of a solution containing Aldol^®^ 495 in a Polypropylene (PP) tube (**C**) and of the PP tube once emptied (**D**). Pictures kindly supplied by Dr Julian Ihssen, Biosynth Carbosynth.

Aldol^®^ substrates allow for the detection of more than one enzyme simultaneously. Aldol^®^ 495 β-D-galactopyranoside **30** is used together with the indoxyl substrate 5-bromo-4-chloro-3-indoxyl-β-D-glucuronic acid **33** (Figure 7), which generates a green-blue colour upon reaction with β-glucuronidase. If both a β-galactosidase and a β-glucuronidase enzymes are present in a bacterial species, dark violet appears as a mixture of both dyes. This allows one to distinguish between *Klebsiella pneumoniae* (β-galactosidase positive and β-glucuronidase negative, orange colour), *E. coli* (β-galactosidase positive and β-glucuronidase positive, dark-violet colour), and *Salmonella enteritidis* (negative for both enzymatic activities, uncoloured) on a single plate (Figure 8A) [49]. Aldol^®^ 495 β-D-galactopyranoside **30** is also used in the detection of *Clostridium perfringens* cultivated under anaerobic conditions, the presence of which can be detected by the formation of a precipitate appearing green fluorescent on a membrane filter (Figure 8B) [49].

The colours in solid chromogenic media are also manipulated by co-adding an Aldol^®^ and an indoxyl substrate with the same enzyme-labile group or two indoxyl substrates with the same enzyme labile group to the same plate. This will result in the development of a new (mixed) colour for positive microbial colonies, which further broadens the available range of colours on agar plates [49].

A special case as a part of this range is Aldol^®^ 495. The fluorogenic enzyme substrates based on Aldol^®^ 495 release upon intramolecular aldol type condensation, 10H-indolo [1,2-a] indole (IO) and (5H,7H)-indolo [1,2-a] quinoline (IQ) [50]. Interestingly, the released IO and IQ show a solvatochromic effect and are strongly fluorescent. IO and IQ signalophors are insoluble in water but soluble in other materials including polypropylene, polyethylene, oils, various solvents and emulsions. The above-mentioned indicators were administered as precursors conjugated to an enzyme substrate and the cleavage by the specific enzyme generated the fluorescent species IO or IQ. Thanks to their hydrophobicity, IO and IQ are able to diffuse and dissolve in oils and Polypropylene/ Polyethylene (PP/PE) plastic materials. Thus, when plastic containers made of or coated with PP/PE are used, IO or IQ are released following the triggering event, diffuse into the solid container and become embedded within the solid latex [50]. The resulting fluorescent emission is therefore measured after the containers are emptied and washed. This property has interesting novel applications. Firstly, the system is convenient when used with cloudy or strongly coloured samples, or with poor optical properties. Secondly, the fluorescence of unstable samples whose shelf life is shorter than the time required for the analysis in the liquid phase can be detected from the solid container. Furthermore, the emission of toxic or dangerous samples that requires careful handling from the operator can be measured in the solid state after emptying, cleaning and disinfecting the containers. An example is shown in Figure 8C, where Aldol^®^ 495 galactoside is used in the presence of purified β-galactosidase. Aldol^®^ 495 is generated by enzymatic cleavage and diffuses in the PP tube, showing green emission even when the tube is emptied (Figure 8D).

Another important feature of Aldol^®^ substrates among others soluble and insoluble substrates is the improved stability of their esters. For example, Aldol^®^ 458 acetate demonstrates minimal unspecific hydrolysis in comparison with much higher hydrolysis rates in indoxyl-, 4-MU- and fluorescein-conjugates [49].

**Scheme 6:**
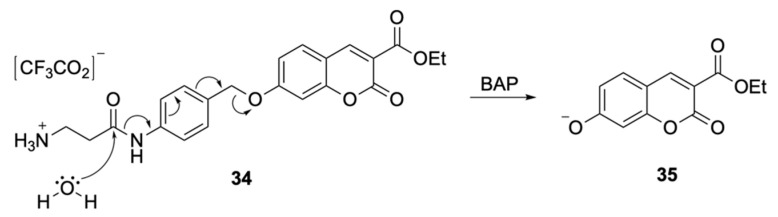
Formation of 3-carbethoxy-7-hydroxycoumarin **34** from fluorogenic substrate **35** [21].

Although some coumarin derivates are water-soluble as previously discussed, others are not and were employed as insoluble fluorogenic enzyme substrates. A recent example was synthesised by Váradi and co-workers [21]. Water-soluble fluorogenic substrate **34** specific for β-alanyl aminopeptidases (BAP) was used for the detection of *P. aeruginosa* in clinical applications. Following hydrolysis of the β-alanine-amide bond by BAP in substrate **34**, a 1,6-elimination and the self-immolative loss of a *p*-aminobenzylidene moiety takes place, releasing insoluble 3-carbethoxy-7-hydroxycoumarin **35** (Scheme 6). Since the pK_a_ of **35** is 7.3, the predominant species at physiological pH is its deprotonated phenoxide form, which is strongly emissive. Substrate **35** allowed for the rapid and effective detection of *P. aeruginosa* with discrimination between the latter and *S. marcescens* [21].

**Scheme 7:**
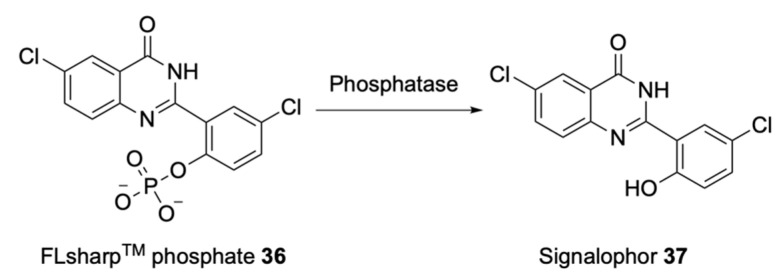
Formation of signalophor **36** by hydrolysis of FLsharp™—Phosphate **37** catalysed by phosphatase [51].

An interesting signalophor used in probes for bacteria detection applications is 6-chloro-2-(5-chloro-2-hydroxyphenyl)-quinazolin-4(3H)-one, also known as ELF^®^, a trademark of Molecular Probes, Inc. Its phosphate derivative **36** is commercialized by Biosynth Carbosynth under the name FLsharp™-Phosphate. It is a water-soluble, non-fluorescent alkaline phosphatase (APase) substrate. Following cleavage by APase, it forms a UV-excited, highly fluorescent yellow-green precipitate **37** (Scheme 7) (optimally excited at 360 nm, with emission at 530 nm) [51]. When ELF^®^ substrates are employed, short detection times of the fluorophore are required. ELF^®^ substrates do not show an inhibitory effect on bacterial growth, even at high concentrations and are used in acidic, neutral and alkaline media, as well as in anaerobic conditions and do not require the presence of auxiliary agents. FLsharp™-Phosphate was incorporated into plating media for the detection of phosphatase activity of *S. aureus* (BCM**^®^** *S. aureus* ELF**^®^**) and MRSA (BCM**^®^** MRSA ELF**^®^**). Enzymatic hydrolysis generates phenol ELF-OH, which forms a solid aggregate with strong emitting properties [51].

#### 2.1.2. Luminogenic Substrates

In almost every modern technique, luminescence-based assays have proven to be superior to chromogenic and fluorogenic methods. The main advantage of luminescence over fluorescence lies in the fact that irradiation by an external light source is not required; thus, the background signal is extremely low and the sensitivity is high [52]. Luminogenic substrates may emit light in response to the presence of a metabolite (e.g., ATP, NAD^+^ and NADH) or in response to the presence of a specific enzyme [53].

##### Bioluminescent Substrates

**Scheme 8:**
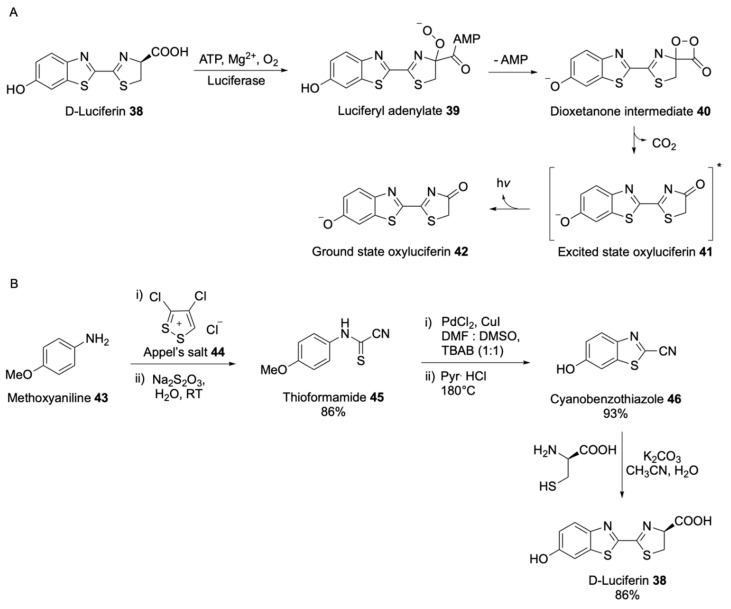
Main pathway of luciferin bioluminescent reaction (**A**); synthesis of d-Luciferin proposed by Prescher et al. (**B**) [58].

Bioluminescence and chemiluminescence are the two main types of luminescence. Bioluminescence is a natural phenomenon occurring in insect fireflies and luminous marine and terrestrial microorganisms [54]. In nature, there are approximately 30 different bioluminescent systems of which only nine were studied to various degrees in terms of their reaction mechanisms [53]. In fireflies, the bioluminescence is produced by an oxidative transformation of luciferin into an excited state intermediate by the enzyme luciferase. The reaction occurs in the presence of oxygen, ATP and Mg^2+^ co-factors. The main pathway of the luciferin bioluminescent reaction is shown in Scheme 8A.

Initially, D-Luciferin **38** reacts with ATP to form luciferyl adenylate **39**. Luciferyl adenylate is then oxidised through a single electron transfer mechanism generating an intermediate peroxide, which further converts into a dioxetanone intermediate **40**. The dioxetanone intermediate decomposes with the concomitant release of CO_2_ and generates the excited state oxyluciferin **41**, which returns to its ground state **42** by emitting light [55,56]. Natural D-Luciferin reacts with luciferase emitting light at 557 nm (green-yellow).

The synthesis of luciferin involves the condensation of cyanobenzothiazole **46** with D- or L-Cysteine. Luciferin analogues are made with the same procedure by starting from a substituted cyanobenzothiazole. The synthesis of intermediate **46** was improved over the years and several procedures are available [57,58,59], the most popular involving the dehydration of a benzothiazole amide, a Sandmeyer reaction using a cyanide source or a Michael condensation with benzoquinone [59]. An interesting synthetic route towards cyanobenzothiazole 46 was reported by Prescher et al (Scheme 8B), starting from the condensation of p-methoxyaniline 48 43 with Appel’s salt 44, to generate thioformamide 45. A subsequent step of cyclisation and deprotection of intermediate 50 generates the cyanobenzothiazole 46. The same authors also described a one-pot procedure towards intermediate **46** starting from aniline **43** and Appel’s salt **44** [58].

The native system of bioluminescence made of luciferase (Fluc) and luciferin (Dluc) presents some limitations for their use in analytical or clinical microbiology. Luciferin Dluc has low tissue permeability, low stability and spontaneous oxidation to dehydro-luciferin, which inhibits the Fluc luciferase. Wild-type luciferase Fluc also has low stability and is inhibited by luciferin metabolites, d-luciferyl-AMP and oxyluciferin, or by damage to the luciferin molecule through a radical mechanism [53].

Chemical modifications of luciferin allowed for a regulated release of the luminescent signal. This feature was further developed by the concept of proluciferin or caged luciferins, which are non-luminescent protected luciferin derivatives containing a masking group capable of preventing the bioluminescence reaction. The masking group needs to be cleaved off the proluciferin/caged luciferin by a specific bacterial enzyme first, and the resulting luciferin derivative exhibits chemiluminescent properties upon exposure to luciferase [60]. Depending on the enzymatic cleavage involved, several proluciferins were designed to contain a labile group attached to the oxygen in DLuc or the nitrogen in ALuc.

**Figure 9:**
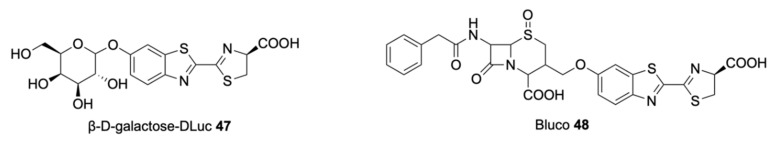
Structures of probes **47** and **48**.

An example is a specific substrate for β-galactosidase in the form of a β-D-galactose-Dluc conjugate probe **47** (Figure 9), which has applications in the detection of coliforms and specifically *E. coli* [61]. In another example, bacterial resistance to β-lactam antibiotics was detected with a β-lactamase-sensitive conjugate of a luciferin-cephalosporin analogue (Bluco) **48** (Figure 9). The bacterial β-lactamase enzyme cleaves the β-lactam ring in the conjugate probe, allowing the negative charge on the nitrogen atom to initiate a cascade of events which terminates with the extrusion of a Dluc molecule, which then reacts with luciferase emitting a measurable signal [61].

Luciferase is an enzyme and an essential element for the generation of a bioluminescent signal. As luciferase dictates the performance of bioluminescent detection, we provide a brief overview of the latest improvements on this protein. As native luciferases present some previously described limitations, genetically modified variants with improved stability properties were developed [53,60]. An example is X-Shining™ Luciferase (patent pending), a genetically engineered luciferase originally from the North-American firefly *Photuris pennsylvanica*, which has strongly improved thermostability and storage stability. The X-Shining™ luciferase in reaction with Dluc luciferin was shown to exhibit excellent signal linearity over a wide ATP concentration range, from 8 fmol to 1 nmol in 96-well plate assays with 0.1 mL liquid volume. This sensitivity is beneficial for microbiological applications, for example, detecting low-level bacterial contamination in samples such as blood, urine and milk. ATP production measured by the luciferin-luciferase assay has also been used successfully to study the effects of antibiotics on bacterial populations [62], and the improved X-Shining™ luciferase increases the sensitivity of these assays.

##### Chemiluminescent Substrates

Chemiluminescence is characterized by the emission of light as the result of a chemical reaction. Chemiluminescence, in contrast to bioluminescence, is a non-enzymatic phenomenon in which a chemical reaction produces a high energy intermediate that returns to its ground state emitting light provided molecular oxygen or hydrogen peroxide is present [54,63,64].

**Scheme 9:**
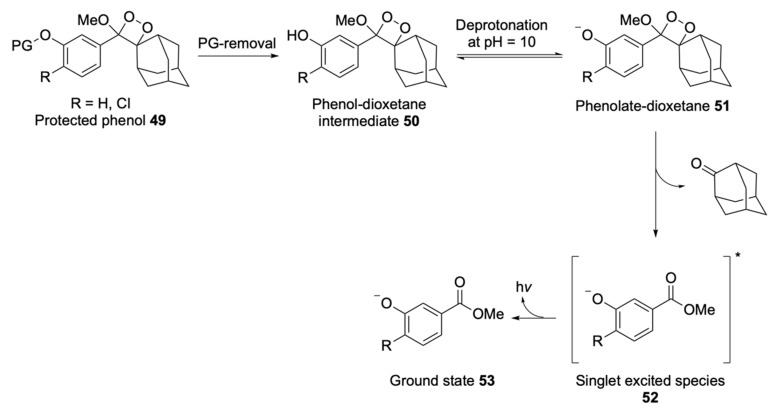
Mechanism of chemiluminescent emission of 1,2-dioxetane **49** [65].

1,2-Dioxetanes as chemiluminescence probes were introduced about 30 years ago by Schaap and generate the luminescent signal by a two-step activation pathway (Scheme 9) [52,65,66]. In the first step, the protected phenol **49** is deprotected by enzymatic activity at physiological pH, generating a phenol-dioxetane intermediate **50**. In the second step, this intermediate is deprotonated by increasing the pH to 10 and the phenolate species **51** triggers the chemiexcitation phenomenon, generating a singlet excited species **52** emitting light which then returns to its energy ground state **53** (Scheme 9).

Although the presence of the adamantanylene moiety does not contribute to the chemiluminescence properties, it confers stability to the dioxetane derivatives resulting in a convenient shelf life. The presence of the methoxy moiety lowers the energy of the singlet excited state. Notably, luciferin and dioxetanes present some structural similarities associated with their emissive properties: both present a four-membered ring peroxide, an activated fluorophore and a trigger [52].

The drawback of these 1,2-dioxetane probes is that luminescence quenching is occurring in water. This is overcome by the addition of surfactants that confer a hydrophobic environment and reduce the quenching. However, the presence of surfactants limits the use for in vivo applications due to their toxicity [52,65,66].

In order to make dioxetanes suitable for in vitro and in vivo applications, the chemiluminescence quenching in water needs to be suppressed and they should act as a single-component molecule probe. The Shabat group developed highly efficient fluorophores by introducing an electron-withdrawing acrylic substituent into the *ortho* position onto the phenoxy-dioxetane moiety, acting as a π-conjugated donor-acceptor. Up to a 3000-fold increase in the chemiluminescence quantum yield in aqueous media was achieved, without the need for surfactants, thanks to an energy transfer mechanism occurring under physiological conditions. These probes emit green light [65,67].

The Shabat group together with Biosynth Carbosynth developed a series of phenolate-dioxetane chemiluminescent enzyme substrates for bacterial detection, commercially available under the name Aquaspark^TM^ [68]. In Aquaspark^TM^ dioxetanes, an enzyme substrate is conjugated to the phenolate, so that the chemiluminescence phenomenon is triggered by the enzymatic cleavage, which is used in the detection of bacteria. The Aquaspark^TM^-based probes offer a comparable or even superior sensitivity compared to Fluc luciferin, with no need for chemical enhancers or additives (like in traditional chemiluminescence). In addition, the chemistry of the new dioxetane probes remains flexible, allowing for the introduction of a number of different substituents to the phenolic group of the probe, making them very versatile tools. In analogy with classic chromogenic substrates, AquaSpark^TM^ molecules can be modified with different enzyme labile groups, which can be cleaved by specific enzymes produced by the bacteria of interest. This way, AquaSpark^TM^ combines two well-known working principles (high sensitivity chemiluminescence and enzymatic release of a specific substrate) for a rapid, specific and cost-effective detection of bacterial species [68].

**Scheme 10:**
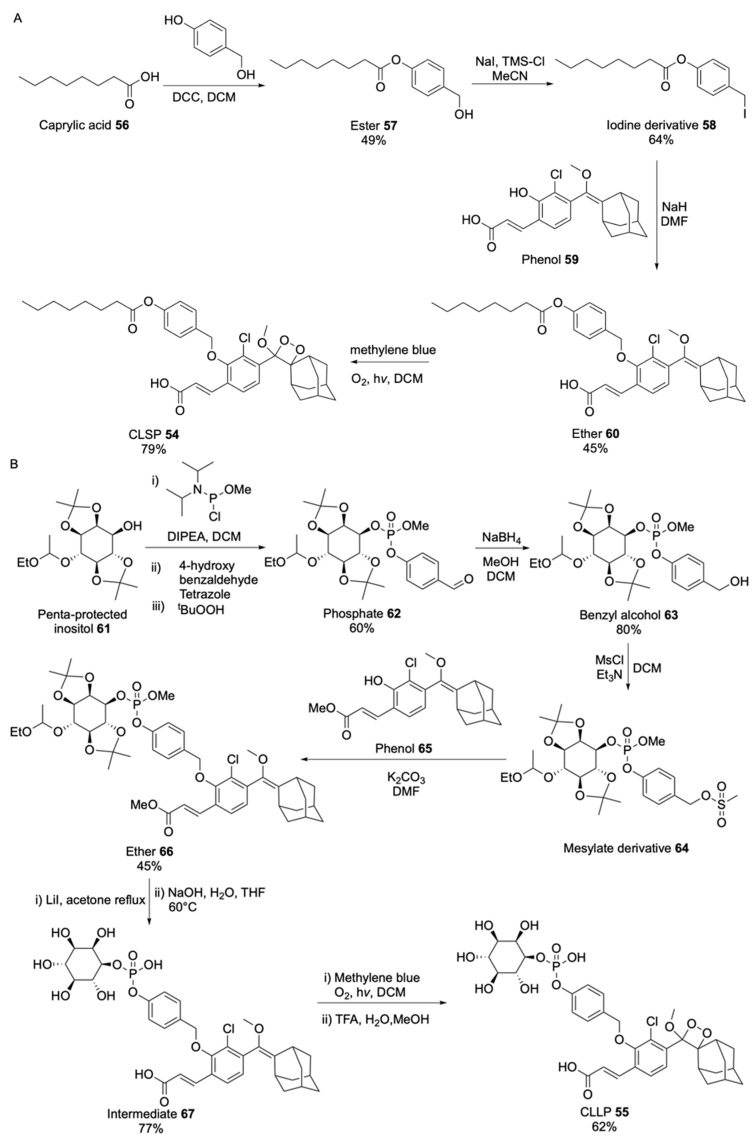
Synthesis of CLSP **54** (**A**) and CLLP **55** (**B**) [69].

Two examples of dioxetane-based enzyme substrates that find applications in the identification of bacteria are CLSP **54** (Scheme 10A) and CLLP **55** (Scheme 10B), used for the detection of *Salmonella* and *Listeria monocytogenes,* respectively [69]. The triggering responsive group within CLSP **54** is a C8 ester, a well-known substrate for *Salmonella* esterase. In CLLP **55**, the triggering group is a myo-inositol 1-phosphate, which is a *L. monocytogenes*-specific substrate for the virulence factor phosphatidylinositol-specific phospholipase C (PI-PLC). The removal of the enzyme-labile group from the substrate by a specific enzymatic interaction and successive spontaneous 1,6-elimination generates the green-emitting phenolate species [69].

The synthesis of CLSP **54** starts from the esterification of caprylic acid **56** with 4-hydroxybenzylic alcohol. Ester **57** is converted into iodine derivative **58**, which reacts with phenol **59** to form ether **60**. The enol ether moiety of ether **60** is finally oxidised with singlet oxygen generating dioxetane CLSP **54** (Scheme 10A) [69].

The synthesis of CLLP **55** starts from the penta-protected inositol **61** and 4-hydroxybenzahldehyde tetrazole obtaining phosphate **62**, in which the aldehyde functionality is reduced to benzyl alcohol in **63**, and subsequently converted into the mesylate derivative **64**. Mesylate **64** and phenol **65** reacts in an S_N_2 reaction generating ether **66**, which is deprotected from the methyl phosphonate protecting group and the methyl ester from the acrylate moiety to form **67**. The last step is an oxidation of intermediate **67** with singlet oxygen, which results in dioxetane CLLP **55** (Scheme 10B) [69].

**Figure 10:**
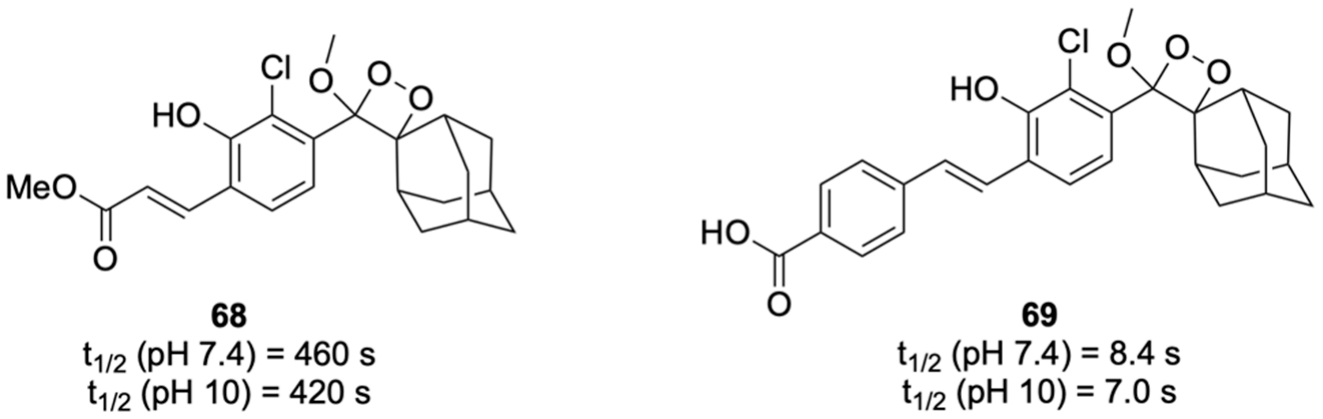
Structures of improved luminophores **68** and **69** [65].

The rate of chemiexcitation of the phenolate-dioxetane upon deprotection highly influences the sensitivity of the analytical bioassay since the signal/background rate is higher when photons are released within a short period of time [64]. Therefore, probes where a faster chemiexcitation occurred were designed by including a styryl moiety in place of the acryl one, which chemiexcitation showed to be two orders of magnitude faster due to the formation of a stabilised phenoxyl radical. This is demonstrated by the half-life of the luminophores **68** and **69 (**Figure 10). The half-life of the acryl derivative **68** is more than 50 times higher than the styryl derivative **69** at physiological pH and around 60 time higher at pH = 10 [65].

**Scheme 11:**
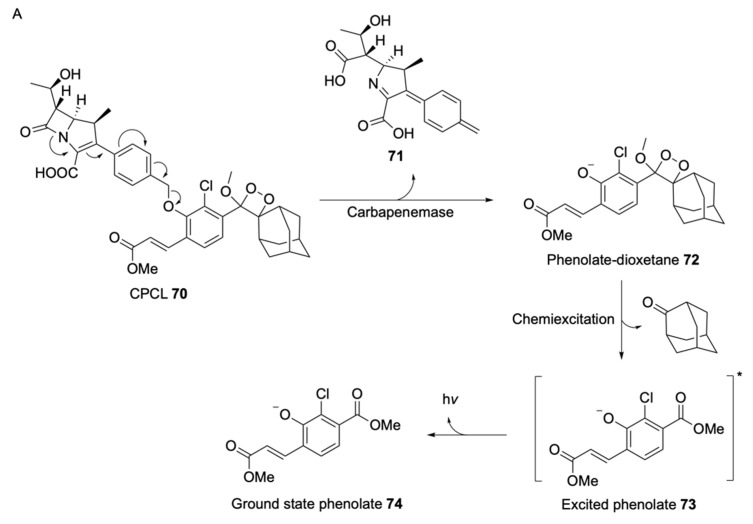

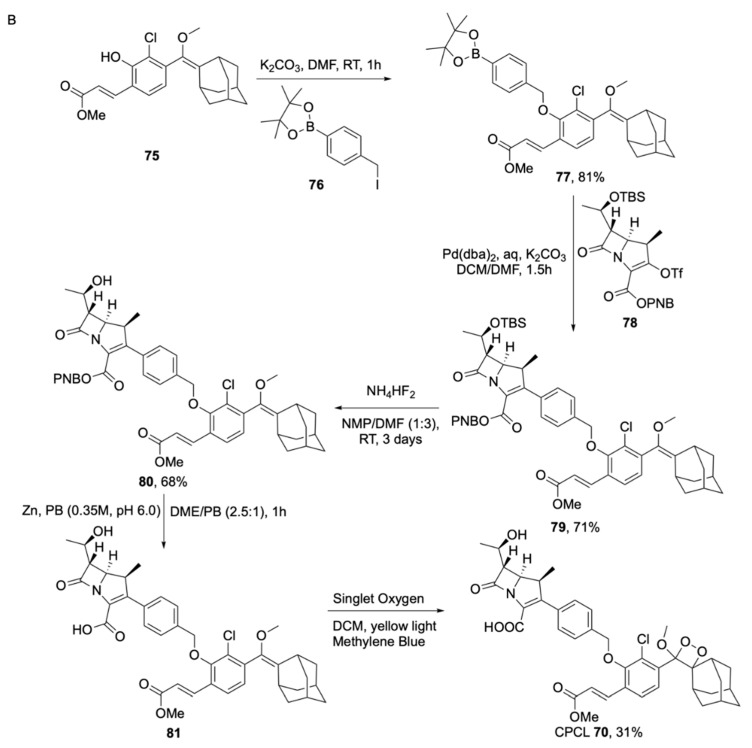
Mechanism of action (**A**) and synthesis (**B**) of dioxetane-based enzyme substrate CPCL **70** [70].

A third example of a dioxetane-based enzyme substrate with a role in microbiology is CPCL **70** (Scheme 11) [70], a probe containing a β-lactam core that is used for the detection of the carbapenemase enzyme in bacteria. The CPCL method demonstrates proof of principle for the rapid screening of bacterial resistance and susceptibility to antibiotics, which could bring significant benefits for the prescription of effective antibiotic therapies to patients. Carbapenemase-producing organisms (CPOs) are a threat to the current and future efficacy of carbapenem antibiotics due to their acquired antibiotic resistance. Therefore, carbapenem antibiotics should be administered carefully in the presence of a pathology caused by CPOs. For this reason, fast and reliable detection methods of CPOs are crucial [70].

In CPCL, the dioxetane-base luminophore is attached to a carbapenemase-recognisable core through a 4-hydroxymethylphenyl linker. The carbapenemase-mediated enzymatic hydrolysis of the β-lactam core is followed by a spontaneous 1,8-elimination of carboxylic acid **71**, generating phenolate-dioxetane **72**, which rapidly generates phenolate species **73** in a chemiexcitation process. Finally, phenolate **73** decays from its excited state to the ground state species **74** by emitting a photon in the green region (Scheme 11A) [70].

The synthesis of CPCL probe **70** starts from a nucleophilic substitution of enol ether **75** with iodide **76** to generate boronate ester **77**. This undergoes Suzuki coupling with triflate **78**, forming intermediate **79**, which is TBS and PNB deprotected in two steps to generate CPCL **70** (Scheme 11B) [70].

#### 2.1.3. Electrogenic Substrates

Following enzymatic activity, electrogenic substrates produce electrical potential associated with a current which is measured with an electrode. The potential benefit of electrogenic substrates are miniature enzyme sensors, which may allow for the drastic reduction in the cost of assays and replace expensive optical equipment with inexpensive electronics [71]. Recent publications have suggested that the use of electrogenic substrates for bacterial detection is a promising strategy in microbiology [71,72].

**Scheme 12:**
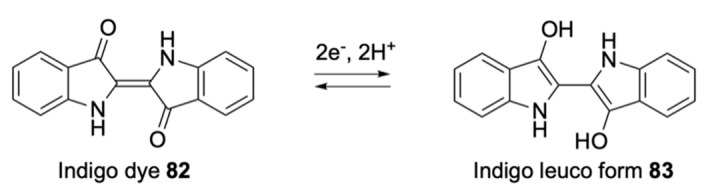
Reduction of indigo **82** to its leuco form **83**.

Indoxyl substrates such as X-Gal are also used as electrogenic substrates. In this case, indigo **82** is electrochemically converted to its colourless leuco form **83** (Scheme 12). It is used in electrogenic enzyme assays to monitor the indigo formed by oxidative dimerization of the indoxyl moiety [73,74]. Indigo, which precipitates on the surface of an electrode, is detected by electro-reduction of the indigo dye to its leuco form. The amount of indigo formation correlates to the current flow at a given potential. Indoxyl-based electrochemical assays focus on DNA hybridisation [75,76], combined with indirect ELISA techniques. Indirect ELISA assays use enzymes, such as alkaline phosphatase, as markers that are attached to secondary antibodies that will react with suitable enzyme substrate indicators. However, rather than using a chromogenic, fluorogenic or luminogenic substrate to visualize the marker enzyme activity, electrochemical methods are used instead [75,76,77].

All examples of this principle are limited to different types of ELISA set ups. The reason is that ELISAs use enzyme-tagged antibodies for the purpose of signal amplification. Just one single antibody will produce thousands of equivalents of indigo. This signal amplification is needed because the detection of indigo precipitates on the electrodes is not very sensitive. The problem of insensitivity does not lie with electrochemistry, but with the process of precipitation itself. In effect, the amplification caused by the enzyme that is linked to the antibodies is lost by the lack of efficiency in indigo detection [73,74].

Using 3-indoxyl phosphate (3-IP) and silver ions as a substrate of alkaline phosphatase a genosensor device was developed for the detection of *S. pneumoniae* [77]. In this approach, a biotinylated DNA probe was immobilized onto a screen-printed carbon electrode (SPCE). The hybridized DNA was subsequently tagged with an anti-fluorescein alkaline phosphatase-labelled antibody that is able to catalyse 3-IP into an indigo blue product. This indigo blue product reduced silver ions into metallic silver, which are electrochemically measured by anodic stripping voltammetry [77].

To increase sensitivity, electrogenic substrates are attached to the surface of an electrode. For this purpose, thiol anchors are connected to the substrates that bind to gold surfaces. This principle was extensively demonstrated using hydroquinone electrogens by Mrksich and coworkers [78,79]. The authors observed that the rates of enzymatic hydrolysis of gold immobilized ester substrates can be measured in real time voltametrically with no adverse effect on the assay caused by immobilization. This proves that this concept is suitable for the sensitive detection of specific enzymes or groups of enzymes in solutions. However, this approach is not applicable to indoxyl substrates due to the requirement of indoxyl dimerization for indigo formation, which is not achievable if immobilized on electrodes. In another innovative approach, the reporter enzyme alkaline phosphatase (AP) was introduced/overexpressed in the target microorganisms (in this case *E. coli* TG1) via a phagemid [80,81], using *p-*aminophenyl phosphate as a substrate. This was previously reported to be an excellent substrate for AP in electrochemical assays [82]. The porosity of the cell wall allows *p-*aminophenyl phosphate to passively diffuse into the periplasmic area of *E. coli* where the AP is present. The product of the reaction, *p*-aminophenol (*p*-AP), diffuses out and oxidizes at the working electrode [81,83,84]. The authors claimed outstanding detection ability of 1 CFU/mL from only 50 mL of contaminated water sample within 2–3 h [80].

### 2.2. Classification Based on Enzyme Triggering the Signal

In biological assays, the activity of a marker enzyme is used to detect or measure the presence of bacteria in a sample. Thus far, in this review the enzyme substrates used for the detection of bacteria are classified based on the properties of the signalophor. Another possible classification is based on the type of enzyme triggering the signal; these are either hydrolytic or non-hydrolytic.

#### 2.2.1. Hydrolytic Enzymes

The enzymes most commonly used for bacterial detection are hydrolytic enzymes such as alkaline phosphatase, β-galactosidase, β-glucuronidase, β-glucosidase that act on phosphoric acid, galactoside, glucoside and glucuronide substrates, respectively [1,2,3,4]. These substrate molecules are derivatized at their hydroxyl moieties with either chromogenic, fluorogenic, or chemiluminescent signalogens for a variety of applications, and are mainly discussed in Section 2.1 [1,2,3,4,41]. Hydrolytic cleavage of the sugar moiety from the aglycone is mediated by the relevant hydrolytic enzyme resulting in the liberation of a signalogenic hydroxyaryl derivative [41]. Common substrates for hydrolytic enzymes are typically esters or glycosides of phenolics and are not fully stable under the conditions of enrichment. This lack of stability in aqueous environments is manifested by a degree of non-specific hydrolysis which is unrelated to the presence of microbial activity. This creates an undesirable background signal, which could lead to false positive results if no appropriate controls are used. Moreover, these substrates are not very specific for the target pathogens and are indeed indicators for common hydrolases. This lack of specificity for microbial substrates further adds to the high number of false positive results and limits their use as a biomarker [85]. Conrad and He invented a new class of fluorogenic substrates including derivatives of halo-pyrene-disulfonic acids that are highly stable to non-enzymatic hydrolysis, thus providing high sensitivity and a broad dynamic range [86]. In addition, the substrates have high solubility in aqueous media, longwave excitation and high Stokes’ shifted emission wavelengths, which is used in fluorometric assays. In another study, a series of fluorescein esters with different chain lengths were evaluated for their effect on spontaneous and the enzymatic hydrolyses [87]. The authors proposed that fluorescein dilaurate is a better substrate for the fluorometric assay of lipases [87].

#### 2.2.2. Non-Hydrolytic

Certain metabolic enzymes are responsible for substrate degradation. These metabolic enzymes are specific for some bacterial species, genii or families, and can therefore be considered biomarkers that provide information about the activity and growth of the organism and detect the metabolic states of living cells. Non-hydrolytic substrates refer to derivatives in which the signalogen is generated from the substrate via non-hydrolytic enzymatic activity. Examples include redox substrates, where a signalophor is generated by enzymatic oxidation or reduction. These include tetrazolium-formazan substrates and resazurin substrates.

**Scheme 13:**
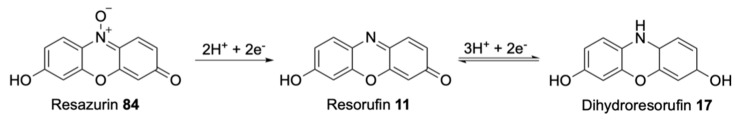
Reduction of resazurin **84** to resorufin **11** and further to dihydroresorufin **17**.

**Scheme 14:**
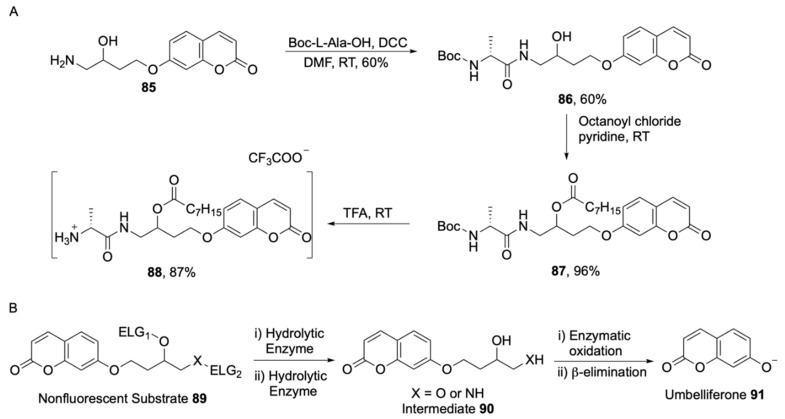
Synthesis of Boolean substrate **88** (**A**) and mechanism of action of general Boolean substrate **89** (**B**).

The strongly fluorescent pink resorufin **11** can also be produced via the reduction of weakly fluorescent blue resazurin **84** (Scheme 13) by aerobic respiration in metabolically active cells7 [88]. Resazurin solutions are marketed under the trade name alamarBlue as indicators of cell health for a number of applications, measuring viability. As a redox indicator, it is used as an alternative to 3-(4,5-dimethylthiazol-2-yl)-2,5-diphenyltetrazolium bromide MTT in applications with human and animal cell lines, bacteria and fungi [88]. Resorufin can be further reduced to non-fluorescent dihydroresorufin **17** in a reversible reaction (Scheme 14B). Using Gram-positive and the facultative anaerobic bacterium *Enterococcus faecalis* as a model organism, it was shown that resazurin reduction to resorufin by living cells can only happen intracellularly [89].

Other examples of commonly used non-hydrolytic enzymes in microbiology include luciferases, horse radish peroxidase (HRP) and nitroreductases. Luciferases were discussed earlier in “Bioluminescent Substrates” in Section 2.1.2. HRPs are commonly used for both histochemistry and ELISAs using colorimetric and chemiluminescent substrates. Fluorogenic peroxidase substrates, however, are not sufficiently stable in aqueous solution, which limits their use in similar application [90].

Nitroreductases (NTRs), which reduce nitro groups to amines, are widespread in the microbial world and are therefore used as a universal means of detection [91]. James et al. used 7-nitrocoumarin derivatives as substrates to successfully detect 30 pathogenic microbial strains, including bacteria and yeast [91]. The sensitivity is stronger with methyl 7-nitrocoumarin-3-carboxylate when compared to other 7-nitrocoumarins [91]. The end product of this substrate is water soluble, unaffected by heat, highly stable and intensely fluorescent. Zhang et al. developed a nitroreductase (NTR) responsive fluorescent probe (NRFP) with a fluorescence off–on feature for identifying *Listeria* in vitro and in vivo with high specificity and sensitivity and low cytotoxicity [92]. Using an NRFP system, the author achieved excellent specificity to NTRs compared to other reductants and a rapid response time of 10 mins. Moreover, NRFP is used to distinguish between *Listeria* and other bacteria based on NTR expression profiles [92].

#### 2.2.3. Boolean Substrates

Wick et al. [85] disclosed a versatile formula for making microbial probes, which can be considered both hydrolytic and non-hydrolytic (Figure 10) after discovering that microbial metabolization of 1-aryloxy-3-propanols is associated with the release of phenols (ArOH). This occurs because the 3-hydroxyl group in the side chain of the phenolic ether is readily available to enzymes such as dehydrogenases, which are able to oxidize such hydroxyl group to form a carbonyl moiety. The resulting carbonyl derivative undergoes β-elimination, generating and releasing the above-mentioned phenol [85]. This process involves the cleavage of a thermodynamically favourable C-O bond, which is inert towards hydrolysis under the conditions used in the incubation or enrichment [84]. The aryloxy group of 1-aryloxy-3-propanol was shown to act as a suitable signalogen with fluorogenic, chromogenic, luminogenic or electrogenic properties. The authors disclosed a number of derivatives based on this compound and described their respective application in bacterial detection/identification [85].

**Figure 11:**
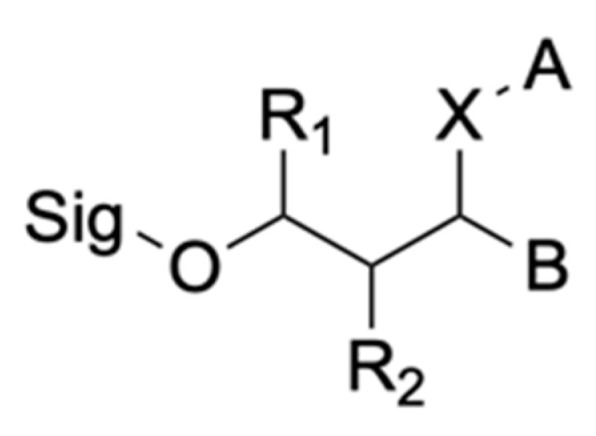
Chemical structure of the Boolean substrate, where A is cleaved by the first hydrolytic enzyme, B is cleaved via second hydrolytic enzyme and Sig is eventually released via a non-hydrolytic enzyme.

Interestingly, the formula is used to derivatize Boolean substrates (Figure 11) in which two enzymes are required to generate a signal. In this case, A and B are replaced by two different enzymatic labile group and the signal is not generated until both groups are enzymatically cleaved off [85].

An example of a Boolean substrate is substrate **88** (Scheme 14A), where an ester is used to mask the hydroxyl group and an acyl group is used to mask the amine, which is highly selective towards *Salmonella* spp. This probe was specifically designed considering both the C8-esterase and L-aminopeptidase activities of *Salmonella* spp. Both enzymatic hydrolyses generate substrate **85** (Scheme 14A), which is an efficient fluorogenic substrate for *Enterobacteriacae*, including *Salmonella* spp. This approach shows that masking the reactive moieties with enzyme labile groups provides a useful feature that narrows down the substrate microbial susceptibility, generating a detecting signal only if two or more enzymes are involved in the substrate transformation. The synthetic procedure of substrate **88** is reported in Scheme 14A. The peptide coupling of amine **85** with Boc-L-Ala-OH forms amide **86**, which is esterified to ester **87** in presence of octanoyl chloride and pyridine. Final removal of the Boc protecting group generates substrate **88**.

The mechanism of action is reported in Scheme 14B. The fluorogenic reporter, in this case umbelliferone, is attached to two enzyme labile groups (ELG1 and ELG2) through a spacer group (nonfluorescent substrate **89**). This design increases the stability of the probe towards non-enzymatic hydrolysis vs. substrates, where the ELG is directly attached to the umbelliferone fluorophore. Cleavage of the 2 ELG in two steps catalysed by hydrolytic enzymes generates intermediate **90**, which undergoes enzymatic oxidation and successive β-elimination, releasing the fluorescent reporter umbelliferone. In cases where the bacteria are unable to cause the enzymatic oxidation of intermediate **90**, and therefore cannot release the reporter *via* β-elimination, the chemical oxidant NaIO_4_ is added. This approach is known as CLIPS-O^TM^ technology, owned by Proteéus SA, France and commercialized by Biosynth Carbosynth.

## 3. Emerging Trends for Enzyme-Based Bacterial Detection

The fast, reliable and high-throughput detection of bacterial strains and their metabolites is of high relevance in the diagnostic industry, modern research, environmental testing and food testing [1,2,3,4,93].

Recent developments in modified substrates, combined with advances in microfluidics and electronics are paving the way towards Point of Care (POC) testing devices, which can be used at a patient’s bedside and provide results instantly or in a reasonably short time. POC diagnostic devices provide simplified sample preparation and a low analysis time, while also offering high sensitivity and selectivity at a lower cost compared to centralized lab technologies [93,94,95,96,97]. An example of a qualitative POC device is a diagnostic test for bacterial vaginosis, caused by *G. vaginalis*. A POC device based on a chromogenic substrate [98] was improved by the adoption of a bioluminescent substrate for a marker enzyme, sialidase [99]. In this assay, firefly luciferin conjugated to a sialic acid moiety was used to detect sialidase activity in a real-time assay, aiding the diagnosis of bacterial vaginosis. This improved bioluminescence-based test for bacterial diagnosis has increased sensitivity and is suitable for quantitative measurements. In the future, this test may therefore be used to stratify bacterial vaginosis patients according to different levels of sialidase activity, which may in turn be considered for assessing other risks associated with this infection. This strategy is also useful for monitoring the efficacy of an antibiotic therapy, as the levels of sialidase activity decrease if antibiotic treatment is effective [99].

Lab-on-chip (LOC) is another recent advancement, which brings together fluidics, electronics, optics and biosensors. LOC is being integrated into POC devices as it offers even better diagnostic speed, low cost, ergonomics and sensitivity. An LOC device integrates several laboratory functions into one small platform, typically only mm or cm in size, which makes it a perfect medium for portable devices [100,101]. The incorporation of signalogenic enzyme substrate derivatives within LOC devices is an appealing field, since it would combine the high sensitivity and selectivity of a specific enzymatic reaction associated with a certain bacterial species with fast, economic and portable detecting systems that can be easily used by non-specialised personnel.

In summary, tests for bacterial detection are currently being miniaturized to become more portable and optimized regarding sensitivity, temperature stability and cost effectiveness. This creates an increasing demand for modified signalogenic enzyme substrates. Synthetic chemistry will need to develop further to improve the structural design and allow for the development of new combinations of enzyme substrates with different signalophors. This is crucial to allow for an extended choice of specific substrates and a wider range of marker enzymes, as well as achieving multiple bacterial markers to be detected in a single assay or a POC diagnostic device.

## Figures and Tables

**Table 1:** Key features of modified substrates.

Signalophor	Key Features	Drawbacks
**Chromogenic and Fluorogenic Substrates**		
Nitrophenol	Soluble, chromogenic, yellow	Colour reduction at acidic pH, yellow colour overlapping with growth media
4-Methylumbelliferone (4-MU)	Soluble, fluorogenic, low toxicity, easy hydrolysis	4-MU esters have limited stability in water, blue emission overlaps with media fluorescence, relatively high pK_a_ of 7.8
7-Amino-4-methylcoumarin (7-AMC)	Soluble, blue fluorescence, low toxicity, easy hydrolysis	Low fluorescence at low pH, signal declines over time
7-Hydroxycoumarin-3-carboxylate (EHC)	Higher fluorescence at lower pH, lower toxicity compared to 4-MU	-
Resorufin	Soluble, sensitive, bright pink, relatively low pK_a_ of 6.0	Necessary presence of oxygen to avoid bleaching
Fluorescein	Soluble, sensitive, bright green fluorescence, biocompatible	Low photostability, hydrophilic
Dihydroxynaphthalenes	Insoluble, chromogenic, brown	Require presence of metal ions to form chelates
Indoxyl	Intense tuneable colour, insoluble, used in combination for multiple bacterial detection, versatile	Toxic to some bacterial species, limited to aerobic conditions
Aldols^®^	Chromogenic and fluorogenic properties, tuneable colour, insoluble (except one derivative), can be used in pairs for multiple bacterial detection, versatile, esters are stable, some soluble in plastic	-
ELF^®^	Insoluble, fluorogenic, used in anaerobic conditions, no need for auxiliary agents	-
**Luminogenic Substrates**		
Pro-Luciferins-Luciferase	Bioluminogenic, highly sensitive, soluble	Low tissue and cell membrane permeability, low lipophilicity, inhibitory effects from intermediates
Luminol	Chemiluminescent, blue, soluble, broad application, low cost, ease of usage	Quenches with reducing agents, interference from metals such as Fe, Cu, Mn, and Co, dependent on signal enhancer, intensity reduction at lower pH, non-specific chemiluminescence as result of non-enzymatic hydrolysis
Dioxetanes	Chemiluminescent, stable up to several years at room temperature, highly sensitive	Low cell membrane permeability
**Electrogenic Substrate**		
Indoxyl	Indigo based, colourless	-
*p*-Aminophenol	Effective with phosphatases	-
**Redox Substrates**		
Formazan	Purple, formed by reduction of tetrazoles such as MTT. Used in viability assays	-
Resorufin	Bright pink, formed by reduction of resazurin	Possible further reduction forms colourless dihydroresorufin

**Table 2:** Aldol^®^ products key features.

Aldol^®^	Dye Solubility (Water)	Colour	Fluorescence
458	Soluble at pHβ6	Yellow	Good in aqueous media (green)
470	Insoluble	Yellow	Medium (yellow)
484	Insoluble	Orange-red	-
495	Insoluble	Orange	Good in hydrophobic matrices (green to yellow)
515/518	Insoluble	Red	Good in hydrophobic matrices (red)
